# A Weighted Two-Level Bregman Method with Dictionary Updating for Nonconvex MR Image Reconstruction

**DOI:** 10.1155/2014/128596

**Published:** 2014-09-30

**Authors:** Qiegen Liu, Xi Peng, Jianbo Liu, Dingcheng Yang, Dong Liang

**Affiliations:** ^1^Department of Electronic Information Engineering, Nanchang University, Nanchang 330031, China; ^2^Paul C. Lauterbur Research Centre for Biomedical Imaging, Institute of Biomedical and Health Engineering, Shenzhen Institute of Advanced Technology, Chinese Academy of Sciences, Shenzhen, Guangdong 518055, China; ^3^Shenzhen Key Laboratory for MRI, Chinese Academy of Sciences, Shenzhen 518055, China

## Abstract

Nonconvex optimization has shown that it needs substantially fewer measurements than *l*_1_ minimization for exact recovery under fixed transform/overcomplete dictionary. In this work, two efficient numerical algorithms which are unified by the method named weighted two-level Bregman method with dictionary updating (WTBMDU) are proposed for solving *l_p_* optimization under the dictionary learning model and subjecting the fidelity to the partial measurements. By incorporating the iteratively reweighted norm into the two-level Bregman iteration method with dictionary updating scheme (TBMDU), the modified alternating direction method (ADM) solves the model of pursuing the approximated *l_p_*-norm penalty efficiently. Specifically, the algorithms converge after a relatively small number of iterations, under the formulation of iteratively reweighted *l*_1_ and *l*_2_ minimization. Experimental results on MR image simulations and real MR data, under a variety of sampling trajectories and acceleration factors, consistently demonstrate that the proposed method can efficiently reconstruct MR images from highly undersampled *k*-space data and presents advantages over the current state-of-the-art reconstruction approaches, in terms of higher PSNR and lower HFEN values.

## 1. Introduction

Fast acquisition is an important issue in magnetic resonance imaging (MRI) for avoiding physiological effects and reducing scanning time on patients. Acquisition time is proportional to the number of acquired* k*-space samples [1]. Unfortunately, accelerating acquisition by reducing* k*-space samples leads to noise amplification, blurred object edges, and aliasing artifacts in MR reconstructions. Then improving reconstruction accuracy from highly undersampled* k*-space data becomes a complementary tool to alleviate the above side effects of reducing acquisition.

To cope with the loss of image quality, some prior information should be used in the reconstruction procedure. One process of introducing prior information in the reconstruction is known as “regularization” [2–11]. Tikhonov regularization, a commonly used method that pursuits reconstructions by a *l*_2_-norm minimization, leads to a closed-form solution that can be numerically implemented in an efficient way [6–8, 11]. Moreover, with the advent of compressed sensing (CS) theory, sparsity-promoting regularization has gained popularity in MRI (e.g., the *l*_1_-based regularization) [1–3, 5, 10, 12]. The CS theory states that sparse, or more generally, compressible signals, incoherently acquired in an appropriate sense, can be recovered from a reduced set of measurements that are largely below the Nyquist sampling rate. Exact reconstruction can be achieved by nonlinear algorithms, using *l*_1_ minimization or orthogonal matching pursuit (OMP) [1–3, 5, 10, 12, 13]. Usually, nonconvex optimization like *l*_*p*_ (0 < *p* < 1) minimization will guarantee a better recovery by directly attacking the *l*_0_ minimization problem [14–16]. Chartrand et al. investigated the nonconvex *l*_*p*_ (0 < *p* < 1) norm optimization as a relaxation of the *l*_0_-norm for MRI reconstruction [17, 18] and showed that it often outperforms the *l*_1_-minimization method. Inspired by viewing *p* as a scale parameter, Trzasko and Manduca [15] developed a homotopic *l*_0_-minimization strategy to reconstruct MR image and generalized the nonconvex *l*_*p*_ (0 < *p* < 1) norm to a family of nonconvex functions as alternatives. Candès et al. presented the iteratively reweighted *l*_1_ minimization (IRL1) algorithm [19], which amounts to linearly minimize the log penalty of the total variation. Wong et al. [16] further incorporated a seminonlocal priority into the homotopic *l*_0_-minimization for breast MRI.

Besides the prior-inducing penalty function, another important issue in CS-MRI reconstruction, is the choice of a sparsifying transform, since one of the important requirements in CS-MRI is that the image to be reconstructed has a sparse representation in a given transform domain. At the start, sparse representation was usually realized by total variation (TV) regularization and/or wavelet transform [1, 12, 13, 15, 20, 21]. As it is known, TV prior assumes the target images consist of piecewise constant areas which may not be valid in many practical MRI applications. Other analytically designed bases, such as wavelets and shearlets, also involve their intrinsic deficiencies [20]. Some advanced approaches were developed to address these issues. Knoll et al. introduced the total generalized variation (TGV) for MR image reconstruction problems [22]. Liang et al. [23] applied the nonlocal total variation (NLTV) regularization to improve the signal-to-noise ratio (SNR) in parallel MR imaging, by replacing the gradient functional in conventional TV using a weighted nonlocal gradient function to reduce the blocky effect of TV regularization. Since fixed bases might not be universally optimal for all images, some methods utilizing sparsity prior under adaptive transform/dictionary were developed recently [24–29]. The sparsity in this framework is enforced on overlapping image patches to emphasize local structures. Additionally, the global dictionary consisting of local atoms (prototype) is adapted to the particular image instance, which thereby provides better sparsity. Our work also adopts the similar concept as a penalty term for MRI reconstruction.

After reviewing two important components in CS, an interesting question is whether a better sparsity-inducing function (e.g., *l*_*p*_ (0 < *p* < 1) norm) combined with a better transform (e.g., adaptive dictionary) will lead to better reconstruction from the same set of measurements. To the best of our knowledge, there is only one paper by Shi et al. preliminarily studying the *l*_0_-approximation sparsity under the learned dictionary [14], where the *l*_0_ penalty is relaxed by the nonconvex minimax concave (MC) penalty. The MC penalty approximates the *l*_0_-penalty by gradually increasing a scale parameter. However, their work focused on image processing and the homotopic strategy they used is computationally demanding [14].

In this paper, we propose a novel method to solve the nonconvex minimization problem in CS-MRI by incorporating the reweighting scheme with our recently developed two-level Bregman method with dictionary updating (TBMDU). An ultimate advantage of our proposed method is that, by incorporating the iteratively reweighted scheme into the two-level Bregman method/augmented Lagrangian (AL) with dictionary updating, the *l*_*p*_-based updating step is conducted in each inner iteration of the AL scheme, and both the minimization of the sparsity of image patches and the data-fidelity constraint can be solved in a unified formalism. The modified alternating direction method (ADM) efficiently solves the model of pursuing the approximated *l*_*p*_ (0 < *p* < 1)-norm penalty. Specifically, by applying iteratively reweighted *l*_2_ or *l*_1_ minimization scheme at each AL inner iteration, the proposed algorithm utilizes adaptive weights to encourage the large coefficients to be nonzero and the small ones to be zero under learned dictionary, hence resulting in more compact and discriminative representation for reconstructed image patches. Numerical experiments show that the performance of the derived method is superior to other existing methods under a variety of sampling trajectories and* k*-space acceleration factors. Particularly, it achieves better reconstruction results than those by using TBMDU without adding any computational complexity.

The rest of this paper is organized as follows. We start with a brief review on TBMDU and iteratively reweighting scheme for *l*_*p*_-minimization in Section 2. Consequently, the proposed method WTBMDU is presented in Section 3. Several numerical simulation results are illustrated in Section 4 to show the superiority of the proposed method. Finally, conclusions and discussions are given in Section 5.

## 2. Theory

Plenty of papers, with the theme of compressed sensing (CS) [2, 3], demonstrated that MR images can be reconstructed from very few linear measurements [1–3, 5, 10, 12]. These approaches take advantage of the sparsity inherent in MR images. The well-known example is the success of TV regularization which implies that MR images can be approximated by those having sparse gradients. Generally, if a sparsifying transform *ψ* can be readily defined, ideally one could choose the estimation with the sparsest representation in *ψ* that still matches the limited measurements. That is, by minimizing transform-domain sparsity-promoting energy *J*(*u*) = ||*ψu*||_0_ that is subject to the data consistency *f* = *F*_*p*_ *u* + *n*, where *n* is noise, it yields

(1) min⁡u J(u)s.t.  ||Fpu−f||2≤ε,

where *u* ∈ *C* ^*N*^ is a vector representing the *N*-pixel 2D complex image. *F*_*p*_ ∈ *C* ^*Q*×*N*^ denotes the partially sampled Fourier encoding matrix and *f* ∈ *C* ^*Q*^ represents the acquired data in* k*-space. $\varepsilon =\sqrt{2}\sigma$ is the standard deviation of the zero-mean complex Gaussian noise, and *σ* is the standard deviation of both real and imaginary parts of the noise. Practically, the *l*_0_ quasi-norm ||·||_0_ in the regularization term of (1) is usually relaxed. In this paper, we propose some efficient algorithms for nonconvex relaxation such as *l*_*p*_ quasi-norm ||·||_*p*_, 0 < *p* < 1 [17, 18].

The choice of the sparsifying transform *ψ* is an important consideration in minimizing functional (1). Besides the fixed transforms such as finite-difference and wavelet transform, modeling image patches (signals) by sparse and redundant representations have been drawing considerable attention in recent years [24]. Considering the image patches set *Ru* = [*R*_1_ *u*, *R*_2_ *u*,…, *R*_*L*_ *u*] consisting of *L* signals, *R*_*l*_ *u* ∈ *C* ^*M*^ denotes a vectored form of the $\sqrt{M}\times \sqrt{M}$ patch extracted from the image *u* of size $\sqrt{N}\times \sqrt{N}$. The sparseland model for image patches suggests that each image patch, *R*_*l*_ *u*, could be sparsely represented over a learned dictionary *D* ^*^ [27]; that is,

(2) $$\begin{array}{c} \alpha_{l}^{*}=\arg\underset{\alpha_{l}}{\min}\left({||\alpha_{l}||}_{0}+\frac{\lambda }{2}{||D^{*}\alpha_{l}-R_{l}u||}_{2}^{2}\right),\;l=1,2,\ldots ,L, \end{array}$$

where *α*_*l*_ stands for the sparse coefficient of the *l*th patch. The combination of sparse and redundant representation modeling of signals, together with a learned dictionary using signal examples, has shown its promise in a series of image processing applications [24–29]. For example, in [25, 26], the authors used reference MRI image slices to train the dictionary and achieved limited improvement. Considering that a dictionary learnt from a reference image would not be able to effectively sparsify new features in the current scan; Ravishankar and Bresler [29] suggested learning dictionary from the target image itself and developed a two-step alternative method to remove aliasing artifacts and noise in one step and subsequently fills in the* k*-space data in the other step.

In the following, to make the paper self-contained, we, respectively, review the TBMDU method for MRI reconstruction and reweighted scheme for solving nonconvex relaxation of the *l*_*p*_-quasi-norm minimization.

### 2.1. The TBMDU Framework for MRI Reconstruction

Recently, we proposed a series of dictionary learning methods [30–32] based on augmented Lagrangian/Bregman iterative scheme [33]. Particularly, by using a two-level Bregman iterative method and the relaxed version of sparseland model (i.e., *J*(*u*) = ∑_*l*_(||*α*_*l*_||_1_ + (*λ*/2)||*Dα*_*l*_−*R*_*l*_ *u*||_2_^2^)) as the regularization term for MRI reconstruction [31], the TBMDU method solves the objective function equation (1) by iterating the following:

(3) uk+1=argmin⁡u{min⁡D,Γ∑l(||αl||1+λ2||Dαl−Rlu||22) +μ2||Fpu−fk||22},fk+1=fk+f−Fpuk+1,

where *D* = [*d*_1_, *d*_2_,…, *d*_*J*_] ∈ *C* ^*M*×*J*^ and Γ = [*α*_1_, *α*_2_,…, *α*_*L*_] ∈ *C* ^*J*×*L*^. *λ* stands for the sparse level of the image patches in the “optimal” dictionary. For many natural or medical images, the value of *λ* can be determined empirically with robust performance in our work. *J* = *K* · *M*, in which *K* measures the degree of the overcompleteness of the dictionary.

The proposed TBMDU method consists of a two-level Bregman iterative procedure, where (3) is the outer-level of the method. On the other hand, the inner-level Bregman iterative is employed to solve the subproblem of sparse representation in (3), that is, adding auxiliary variables *z*_*l*_ to convert the unconstrained problem (2) into a constrained formulation:

(4) min⁡D,αl ||αl||1+λ2||zl||22s.t.  zl=Dαl−Rlu, l=1,2,…,L.

Then split Bregman method/augmented Lagrangian is used to solve problem (4) as follows:

(5) (Di+1,αli+1,zli+1) =argmin⁡D,αl,zl∑l||αl||1+λ2||zl||22+β2||zl+Dαl−Rlu−yliβ||22,

(6) $$\begin{array}{c} Y^{i+1}=Y^{i}+\beta \left(Ru-Z^{i+1}-D^{i+1}\Gamma^{i+1}\right). \end{array}$$

We minimize the corresponding AL function (5) alternatively with respect to one variable at a time. This scheme decouples the minimization process and simplifies the optimization task. At the dictionary updating step, by updating (5) with respect to *D* along the gradient descent direction and constraining the norm of each atom to be unit [24], we can get the following update rule:

(7) $$\begin{array}{c} D^{i+1}=D^{i}+\xi Y^{i+1}{\left(\Gamma^{i+1}\right)}^{T},\\ d_{j}^{i+1}=\frac{d_{j}^{i+1}}{{||d_{j}^{i+1}||}_{2}},\;j=1,2,\ldots ,J. \end{array}$$

At the sparse coding stage of updating *α*_*l*_, the optimization problem for each *α*_*l*_ is derived:

(8) $$\begin{array}{c} \alpha_{l}^{i,m+1}=\arg\underset{\alpha_{l}}{\min}{||D^{i}\alpha_{l}-b_{l}-\frac{y_{l}^{i}}{\beta }||}_{2}^{2}+2\frac{\lambda +\beta }{\lambda \beta }{||\alpha_{l}||}_{1}. \end{array}$$

By applying the iterative shrinkage/thresholding algorithm (ISTA) [33, 34] (i.e., performing a gradient descent of the first functional term in (8) and then solving a proximal operator problem) and using the immediate variable *y*_*l*_^*m*+1^ to represent the updating scheme compactly (i.e., the formulation *y*_*l*_^*m*+1^ = (*λβ*/*λ* + *β*)(−*D* ^*i*^ *α*_*l*_^*i*,*m*^ + *b*_*l*_ + *y*_*l*_^*i*^/*β*) derived from (6)), it attains the solution of *α*_*l*_ as follows:

(9) $$\begin{array}{c} \alpha_{l}^{i,m+1}=\text{Shrink}\left(\alpha_{l}^{i,m}+\left(\lambda +\beta \right){\left(D^{i}\right)}^{T}\frac{y_{l}^{m+1}}{\gamma \lambda \beta },\frac{\left(\lambda +\beta \right)}{\gamma \lambda \beta }\right), \end{array}$$

where *γ* > eig((*D* ^*i*^)^*T*^ *D* ^*i*^) and *x* = Shrink(*g*, *μ*) = (*g* · max⁡(|*g*| − *μ*, 0))/(max⁡(|*g*| − *μ*, 0) + *μ*) is the solution of *x* = arg min⁡_*x*_||*x*||_1_ + (1/2*μ*)||*x*−*g*||_2_^2^.

The corresponding procedure of TBMDU is summarized in Algorithm 1. Line 10 is the frequency interpolation step for updating the image *u*. AL scheme and ADM applied to the constrained problem result in an efficient two-step iterative mechanism, which alternatively updates the solution *u* and image-patch related coefficients (Γ, *Y*, and *D*). For a more detailed description of the derivations, the interested readers can refer to [31].

### 2.2. Reviews of Reweighted Scheme on Solving Nonconvex Relaxation of the *l*_*p*_ Quasi-Norm

In (1), the *l*_0_ penalty can be relaxed into several tractable alternatives. For example, some nonconvex relaxations such as *l*_*p*_ quasi-norm ||·||_*p*_, 0 < *p* < 1 [17, 18], log penalty [19], and smoothly clipped absolute deviation (SCAD) penalty [35] were usually used. Compared to *l*_1_ norm, *l*_*p*_ function with 0 < *p* < 1 is a smooth relaxation of *l*_0_ and the geometry of *l*_*p*_ ball gives better approximation on sparsity. Recent numerical results showed that adapting nonconvex optimization technique can reduce the required number of measurements for reconstruction [14–16].

Most current approaches for solving nonconvex *l*_*p*_-quasi-norm optimization can be classified into two categories: reweighted *l*_2_ and reweighted *l*_1_[36–41]. The common idea of these methods is to utilize an optimization transfer technique to iteratively surrogate the nonconvex potential function by a convex function [37, 39, 41]. Usually, the convex function is chosen as a local quadratic approximation or linear local approximation. A technique drawing much interest is the iteratively reweighted least squares (IRLS) method which is widely used in compressed sensing and image processing [36, 40]. Another technique closely related to IRLS is the iteratively reweighted norm (IRN) approach introduced by Rodríguez and Wohlberg for generalized total variation functional [42]. The IRN method pursues to minimize the *l*_*p*_ norm *F*(*α*) = (1/*p*)||*α*||_*p*_^*p*^ for *p* ≤ 2 by using a weighted *l*_2_ norm in an iterative manner. At iteration *k* + 1, the solution *α* ^*k*+1^ is the minimizer of

(10) $$\begin{array}{c} G\left(\alpha \right)=\tilde{F}\left(\alpha ,\alpha^{k}\right)=\frac{1}{2}{||\sqrt{W^{k}}\alpha ||}^{2},\;\sqrt{W^{k}}=\mathrm{diag}\left({\left|\alpha^{k}\right|}^{p-2}\right). \end{array}$$

The sequence of solution {*α* ^*k*^} converges to the minimizer of *F*(*α*) as *k* → +*∞*. It has been proven that IRN is one kind of majorization-minimization (MM) method [42], which involves good property of *G*(*α* ^*k*+1^) ≤ *G*(*α* ^*k*^). In order to extend the iterative reweighting approach IRLS to the general nonconvex function, Mourad and Reilly developed the quadratic local approximation of the nonconvex function as follows [39]:

(11) F(αj,l)=φ(|αj,l|)=ϕ(|αj,lk|)+ϕ′(|αj,lk|)(|αj,l|−|αj,lk|)+ζ0(|αj,l|−|αj,lk|)2,

where *ζ*_0_ ≥ 0 is a suitably chosen parameter and *ϕ*′ is the first derivative of *ϕ*. *j* stands for the *j*th index number of vector *α*_*l*_.

A similar work conducted by Elad and Aharon is the iteratively reweighted *l*_1_ minimization (IRL1) algorithm [27], which iteratively minimizes the linearization of a quasi-logarithmic potential function. Zou and Li [41] presented the linear local approximation of the general nonconvex function and the upper bound of

(12) $$\begin{array}{c} F\left(\alpha_{j,l}\right)=\varphi \left(\left|\alpha_{j,l}\right|\right)=\phi \left(\left|\alpha_{j,l}^{k}\right|\right)+\phi^{\prime }\left(\left|\alpha_{j,l}^{k}\right|\right)\left(\left|\alpha_{j,l}\right|-\left|\alpha_{j,l}^{k}\right|\right). \end{array}$$

Equation (12) aims to approximate the penalty function with its first-order Taylor expansion at the current iteration. Its majorization properties can be easily obtained by exploiting the concavity of the function *ϕ*, which always lies below its tangent. The numerical tests in [17] by Chartrand and Yin demonstrated that IRLS is comparable with IRL1. In [38], the authors presented weighted nonlinear filter for compressed sensing (WNFCS) for general nonconvex minimization by integrating the IRL1 algorithm shown in (12) and their previously developed NFCS framework [43].

## 3. Materials and Methods

As discussed in previous section, dictionary updating and sparse coding to (5) are performed sequentially. Therefore, an interesting question is that will the better sparsity inducing function in the coefficient matrix lead to better image reconstruction under the learned dictionary? In the following, we investigate two variations at the sparse coding step. By employing the *l*_*p*_-seminorms (0 < *p* ≤ 1), it is feasible to expect that utilizing approximations that are closer to the *l*_0_-quasi-norm than *l*_1_ will correspondingly reduce the required number of measurements for accurate reconstruction.

### 3.1. Proposed Method: WTBMDU

By replacing *l*_1_ norm with nonconvex *l*_*p*_ norm, (3) can be rewritten as

(13) uk+1=argmin⁡u{min⁡D,Γ∑l(1p||αl||p+λ2||Dαl−Rlu||22)    +μ2||Fpu−fk||22}.fk+1=fk+f−Fpuk+1.

After some manipulations similar to those in Section 2.1, updating coefficients at the sparse coding stage are deduced as follows:

(14) $$\begin{array}{c} \alpha_{l}^{i,m+1}=\arg\underset{\alpha_{l}}{\min}{||D^{i}\alpha_{l}-b_{l}-\frac{y_{l}^{i}}{\beta }||}_{2}^{2}+2\frac{\lambda +\beta }{\lambda \beta }\frac{1}{p}{||\alpha_{l}||}_{p}. \end{array}$$

When other variables are fixed, numerical strategies are proposed in the following to solve the corresponding nonconvex minimization problems with regard to *α*, by iteratively minimizing a convex majorization of the nonconvex objective function.

#### 3.1.1. Algorithm 1: Reweighed *l*_1_

By inserting the local linear approximation in the sparse coding step, it is possible to transform the nonconvex unconstrained minimization problem into a convex unconstrained problem, which can be solved iteratively. In the penalty functional of (14), like the well-known method ISTA, a proximal operator is employed to approximate the first term, and the reweighted *l*_1_-approximation is employed to the second term:

(15) αli,m+1=argmin⁡αl||Diαl−bl−yliβ||22︸ISTA+2λ+βλβ1p∑j|αj,l|p︸ReL1,

where *j* stands for the *j*th index number of vector *α*_*l*_. Following the definition in (12), where *ϕ*(|*α*_*j*,*l*_|) = (1/*p*)|*α*_*j*,*l*_|^*p*^ and *ϕ*′(|*α*_*j*,*l*_|) = 1/(|*α*_*j*,*l*_|^1−*p*^ + *ε*), it attains the solution of *α*_*l*_:

(16) αli,m+1=argmin⁡αl  γ||αl−[αli,m+(λ+β)(Di)Tylm+1γλβ]||22 +2λ+βλβ||Wli,mαl||1=Shrink(αli,m+(λ+β)(Di)Tylm+1γλβ,(λ+β)Wli,mγλβ),

where *W*_*j*,*l*_^*i*,*m*^ = 1/(|*α*_*j*,*l*_^*i*,*m*^|^1−*p*^ + *ε*), *j* = 1,2,…, *J*. *ε* > 0 is a small parameter to prevent division by zeros.

#### 3.1.2.  Algorithm 2: Reweighed *l*_2_

Compared to the previous reweighted *l*_1_ strategy, the only difference is that the reweighted *l*_2_-approximation in (10) is employed to the second term:

(17) αli,m+1=argmin⁡αl||Diαl−bl−yliβ||22︸ISTA+2λ+βλβ1p||αl||pp︸ReL2.

Then it attains the solution of *α*_*l*_:

(18) αli,m+1=argmin⁡αl  γ||αl−[αli,m+(λ+β)(Di)Tylm+1γλβ]||22+λ+βλβ||Wli,mαl||22.

The minimizer of this least-square problem is given by

(19) $$\begin{array}{c} \alpha_{l}^{i,m+1}=\frac{\gamma \alpha_{l}^{i,m}+\left(\lambda +\beta \right){\left(D^{i}\right)}^{T}y_{l}^{m+1}/\lambda \beta }{\gamma +\left(\lambda +\beta \right)W_{l}^{i,m}/\lambda \beta }, \end{array}$$

where *W*_*j*,*l*_^*i*,*m*^ = 1/(|*α*_*j*,*l*_^*i*,*m*^|^2−*p*^ + *ε*), *j* = 1,2,…, *J*. Similar to that in (14), *ε* > 0 is a small parameter to prevent numerical instabilities.

Now, we summarize our proposed method for MRI reconstruction here, which we call* weighted TBMDU*. The detailed description of the proposed method is listed in Algorithm 2. Similar to TBMDU, the proposed* WTBMDU* method alternatively updates the target solution *u*, image patch related coefficients (Γ, *Y*, and *D*), and auxiliary variables. The difference between the plain TBMDU and the weighted TBMDU mainly lies on the weights used in updating the sparse coefficients. In WTBMDU, the weights are obtained by evaluating the function of variables at the solution of the previous step. Specifically, whether the *W*_*j*,*l*_^*i*,*m*^ = 1/(|*α*_*j*,*l*_^*i*,*m*^|^1−*p*^ + *ε*) in (16) or *W*_*j*,*l*_^*i*,*m*^ = 1/(|*α*_*j*,*l*_^*i*,*m*^|^2−*p*^ + *ε*) in (19), the weighting scheme *W*_*j*,*l*_^*i*,*m*^ decreases as the absolute value of *α*_*j*,*l*_^*i*,*m*^ increases, indicating that it penalizes more on the coefficients with small magnitude value. Therefore, this operation strongly encourages the large coefficients to be nonzero and the small ones to be zero. In line 8 for reweighted *l*_1_ and line 10 for reweighted *l*_2_, the formulations denote operating every element in the matrix component-wise. In the following content of this paper, we denote the reweighted *l*_1_ and reweighted *l*_2_ in WTBMDU as* WTBMDU-L1* and* WTBMDU-L2*, respectively.

The strategy we used in WTBMDU is similar to that used in WNFCS [38], that is, combining the penalized proximal splitting strategy and reweighting strategy. The difference is that WNFCS focus on updating *u* in the simple CS domain and the proximal splitting strategy is employed to the Fourier-related data-fidelity term, while our WTBMDU devotes to updating the coefficients in the adaptive dictionary and the proximal splitting strategy is employed to the dictionary-related sparse representation error term. Additionally, we also derive the updating scheme based on reweighted *l*_2_.

### 3.2. Parameter Values, Continuation Strategy, and Algorithm Convergence

The proposed method involves four parameters: *β*, *μ*, *λ*, and *ζ*. The setting of these parameters is similar to that in TBMDU. Firstly, both parameters *β* and *μ* are the Bregman (or augmented Lagrangian) positive parameters associated with the small image patches and the whole image itself, respectively. One is for the overlapping image patches and the other is for the image solution itself. It has been mathematically proven that the choice of the Bregman parameter has little effect on the final reconstruction quality as long as it is sufficiently small [31, 44, 45]. In our work, the smaller the value of the Bregman parameter is, the more iterations the Bregman method need to reach the stopping condition. Moreover, since *β* and *μ* are with different orders of magnitude, we set *μ* ≈ *ρNβ*/*M* in the AL formalism for the balance between various “penalty” terms. Secondly, *λ* stands for the sparse level of the image patches and can be determined empirically. Finally, the step size *ζ* in the dictionary updating stage can be set to be a small positive number, for example, 0.01.

In summary, there is only the parameter *β* that should be carefully chosen in order to enable the efficiency of the algorithm. In our method, by taking advantage of the typical structure of the problem in this paper, we propose a similar rule to that used in [21]. Specifically, we set *β* and *μ* so as to achieve condition numbers *κ*(1 + *W*_*l*_^*k*,*m*^/*γT*_0_) and *κ*(*FF*_*p*_^*T*^ *F*_*p*_ *F* ^*T*^ + (*β*/*μ*)*F*∑_*l*_ *R*_*l*_^*T*^ *R*_*l*_ *F* ^*T*^) that result in fast convergence of the algorithm. Since *T*_0_ = *λβ*/(*λ* + *β*) and *λ* ≫ *β*, *T*_0_ ≈ *β*. Consequently *κ*(1 + *W*_*l*_^*k*,*m*^/*γT*_0_) is a decreasing function of *β*. Choosing *β* such that *κ*(1 + *W*_*l*_^*k*,*m*^/*γT*_0_) → 1 would require a large *β* and accordingly, the influence of the weight *W*_*l*_^*k*,*m*^ diminishes and the solution of *α*_*l*_^*k*,*m*+1^ trends to the least-square solution (*q* = 2). In our experiments, we observed that this phenomenon would result in an “immature” dictionary that is not learned enough. On the other hand, taking *β* → 0 would increase *κ*(1 + *W*_*l*_^*k*,*m*^/*γT*_0_) which makes (1 + *W*_*l*_^*k*,*m*^/*γT*_0_)^−1^ numerically unstable. The same trend also applies to *κ*(*FF*_*p*_^*T*^ *F*_*p*_ *F* ^*T*^ + (*β*/*μ*)*F*∑_*l*_ *R*_*l*_^*T*^ *R*_*l*_ *F* ^*T*^) as a function of *β*/*μ*. We found that empirically choosing *β* such that *E*(*κ*(*W*_*l*_^*k*,*m*^)) ≈ ∑_*l*_ *κ*(*W*_*l*_^*k*,*m*^)/*L* ∈ [2,6] generally provided good convergence speed in all experiments. Additionally, we can estimate *β* in a more practical way. Specifically, since the number of data samples is huge, we can set *E*(*W*_*l*_^*k*,*m*^) ≈ *γT*_0_, where *E*(*W*_*l*_^*k*,*m*^)∈[0.2,1] is a robust estimate. Together with the fact that *γ* ≈ 100, it leads to *β* ∈ [1/500, 1/100]. As for the parameter *μ*, since *κ*(*FF*_*p*_^*T*^ *F*_*p*_ *F* ^*T*^ + (*β*/*μ*)*F*∑_*l*_ *R*_*l*_^*T*^ *R*_*l*_ *F* ^*T*^) = (*μ* + *βω*)/*βω*, where *ω* = 64 when the patch overlapping is *r* = 1, it concludes that setting *μ* ≈ *ρNβ*/*M* such that *κ*(*FF*_*p*_^*T*^ *F*_*p*_ *F* ^*T*^ + (*β*/*μ*)*F*∑_*l*_ *R*_*l*_^*T*^ *R*_*l*_ *F* ^*T*^) = 64*ρ* + 1 ∈ [4,20] is a reasonable choice, under the assumption that the* k*-space data is significantly undersampled (e.g., *ρ* ∈ [5%, 30%]).

As for the convergence, because of the unconvexity and nonlinearity of the problem in the case of updating dictionary, the global solution may not to be found easily like in TBMDU. Nevertheless, our dictionary is updated by a gradient descent of the AL scheme which leads to a monotonic decrease in the cost function. At the sparse coding stage, since WTBMDU merges the reweighted and penalization strategies, it still maintains the descent property of the latter and local minimum must be attained as demonstrated in [38]. Therefore, both the value of the objective function and the norm of the reconstruction difference between successive iterations can be chosen as the stopping criterion. The convergence property of the algorithm will be presented in the numerical section.

## 4. Experiment Results

In this section, we evaluate the performance of the proposed method using a variety of sampling schemes, with different undersampling factors. Sampling schemes used in our experiments include 2D random sampling [15], Cartesian approximation of multishot variable-density spiral sampling [15], Cartesian sampling with random phase encodings (1D random) [1, 15], and pseudo radial sampling [15, 29]. Reconstruction results on simulated MRI data, a complex phantom, and real MRI data were presented. The MR images tested in the synthetic experiments are from in vivo MR scans of size 512 × 512 (many of which are courtesy (2009, American Radiology Services [Online]. Available: http://www3.americanradiology.com/pls/web1/wwimggal.vmg/) and used in [29]), and the real MRI data examples reported here are of size 256 × 256 (except in Figures 6 and 7 where a phantom of size 512 × 512 was used). According to many prior work on CS-MRI [1, 13, 29], the CS data acquisition was simulated by subsampling the 2D discrete Fourier transform of the MR images (except in the second subsection where real acquired data was used). Our proposed method WTBMDU was compared with the leading DLMRI (the code is available in https://netfiles.uiuc.edu/ravisha3/www/DLMRICODE.zip) [29] and TBMDU methods [31], which have been shown to substantially outperform other CS-MRI methods such as LDP (Matlab codes are available in http://www.stanford.edu/~mlustig/) [1], and the zero-filling reconstruction. DLMRI directly solves the *l*_0_-minimization by OMP while TBMDU is devoted to the *l*_1_-induced sparse minimization. In each given example, the parameters for the DLMRI method were set to be default values.

In the experiments, the nominal values of various parameters were set as patch size $\sqrt{M}=6$, the over-completeness of the dictionary *K* = 1 (correspondingly *J* = 36), and the patch overlap *r* = 1; thereby the number of data samples were *L* = 65536 for $\sqrt{N}=256$ and *L* = 262144 for $\sqrt{N}=512$. $\omega ={\left(\sqrt{M}/r\right)}^{2}=36$, *β* = 0.0056, *μ* = *Nβ*/*M*, *ζ* = 0.01, *λ* = 12, and *m* = 3. To avoid dividing by zero, the parameter *ε* used in the weighted matrix *W*_*q*,*l*_^*i*,*m*^ decreased by 2% after each inner iteration with an initial value of 5. The setting of parameters *m* and *λ* is very similar to that in, [31, 32] and not discussed here due to the limit of paper space. Real-valued dictionaries were used for the simulated experiments with real-valued images, where the over-complete discrete cosine transform (DCT) was chosen as the initial dictionary. Complex-valued dictionaries were used for real MR data, where both the real and imaginary parts were the same DCT matrix. The quality of the reconstruction was quantified using the peak signal-to-noise ratio (PSNR (the PSNR is defined as PSNR = 20  log_10_  255/RMSE, where the RMSE is the root mean error estimated between the ground truth and the reconstructed image)) and high-frequency error norm (HFEN) [29]. All algorithms were implemented in MATLAB 7.1 on a PC equipped with AMD 2.31 GHz CPU and 3 GByte RAM.

### 4.1. Reconstruction of Simulated Data (Real-Valued)

**Figure 1:**
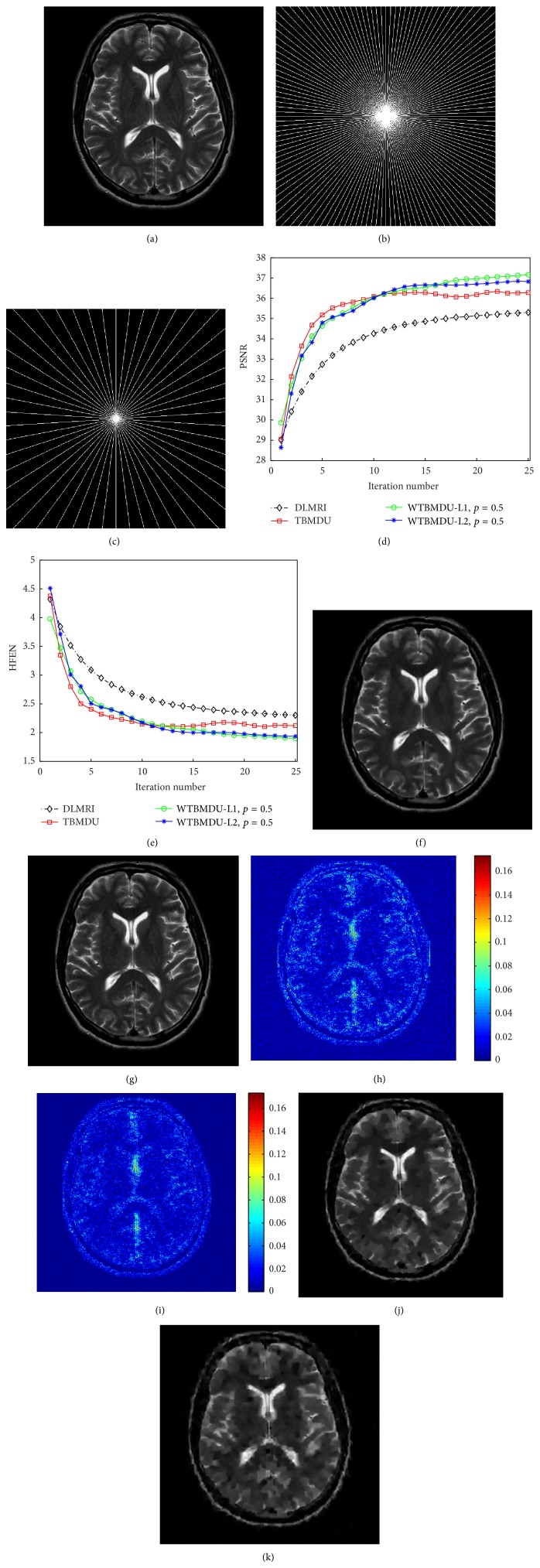
Performance of various algorithms at different undersampling ratios. (a) An axial T2-weighted reference image of the brain. ((b) and (c)) Pseudo radial sampling with 85% and 95% undersampling. (d) and (e) are the PSNR and HFEN versus the number of iterations of DLMRI, TBMDU, and WTBMDU. ((f), (g), (h), and (i)) The reconstruction results and the corresponding error magnitudes of DLMRI and WTBMDU-L1 with *p* = 0.5 at 85% undersampling. ((j) and (h)) The reconstruction results of DLMRI and WTBMDU-L1 with *p* = 0.5 at 95% undersampling.

We first investigate the performance of WTBMDU with the noiseless measurements.  Figure 1 involves an axial T2-weighted reference image of the brain with pseudo radial sampling under 85% and 95% undersampling percentages, respectively (i.e., only acquiring 15% and 5%* k*-space data with corresponding acceleration factors of 6.67 and 20). The plots of PSNR and HFEN values as functions of iteration number under the undersampling percentage of 85% are presented in Figures 1(d) and 1(e). It can be observed that the quantitative measures of both TBMDU and WTBMDU change quickly during the first few iterations. In other words, these measure values only need less iterations to reach the convergence zone and hence the iterative convergence property of our method is better than that of DLMRI. The higher PSNR values and lower HFEN values after convergence also confirm the superiority of our method to DLMRI and TBMDU. The reconstructed results shown in Figures 1(f), 1(g), 1(h), and 1(i) reveal that the method WTBMDU-L1 with *p* = 0.5 provides a more accurate reconstruction on image contrast and sharper anatomical depiction. Compared to DLMRI, the magnitude images of the reconstruction error shown in Figures 1(h) and 1(i) indicate that our method exhibits crisper reconstruction of object edges (the large anatomical structure in the middle region) and preserves finer texture information (the gray matter regions in the bottom-right of the reconstruction). In general, our proposed method provides better intensity fidelity to the fully sampled image.

More obvious differences in visual quality can be observed in the case of 95% undersampling as shown in Figures 1(j) and 1(k). The obtained PSNRs of DLMRI and WTBMDU-L1 with *p* = 0.5 are 26.30 dB and 28.13 dB, respectively. The DLMRI reconstruction in Figure 1(j) based on k-SVD dictionary updating and greedy pursuit of coefficients shows a large number of spurious oscillations, although it gives much improvement than zero-filling and LDP (not shown in this paper). In contrast, WTBMDU shown in Figure 1(k) results in much fewer spurious oscillations and better preservation of edges through iterative weighting of the coefficients under the data-adaptive dictionary. This is especially noticeable for the brain's fissures with sharper anatomical edges.

**Figure 2:**
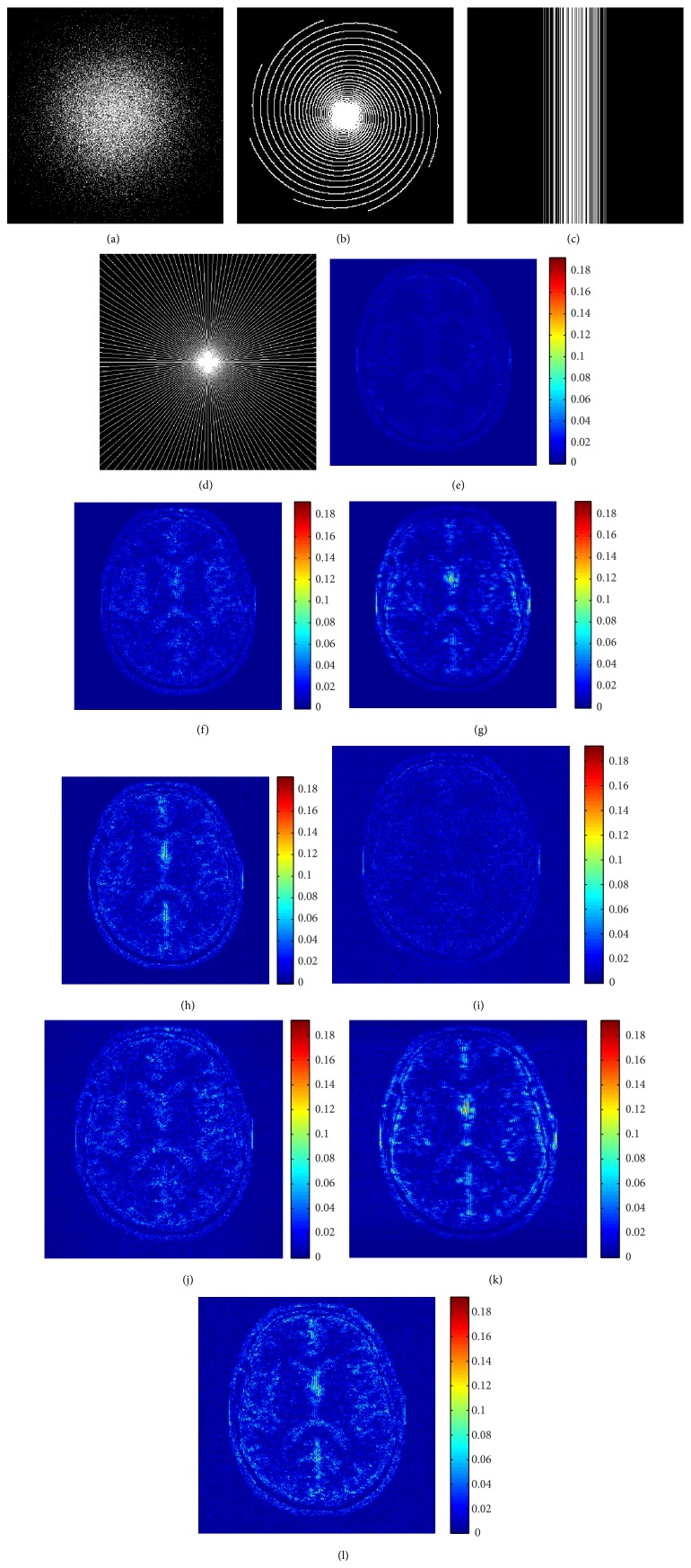
Performance of algorithms at different sampling trajectories with the same undersampling ratio. The first line ((a), (b), (c), and (d)): the variable density 2D random sampling, Cartesian approximation of multishot spiral sampling, Cartesian sampling, and pseudo radial sampling with 86% acceleration factor, respectively. The second and third lines are the reconstruction errors of DLMRI and WTBMDU-L1 with *p* = 0.7 for the four sampling masks.

Figure 2 compares the results generated by DLMRI and WTBMDU using four sampling trajectories roughly under the same undersampling percentage: variable density random with 87% undersampling [15], Cartesian approximation of multishot spiral with 86% undersampling [15], 1D Cartesian trajectory [29], and pseudo radial sampling with 86% undersampling (i.e., 7.11-fold acceleration). The test image is the axial T2-weighted brain image shown in Figure 1(a). As can be observed from the error images, WTBMDU-L1 performs better in reducing aliasing artifacts and maintaining fine details than DLMRI with all verified sampling trajectories.

Table 1 lists the PSNR values of the axial T2-weighted brain image at different sampling trajectories with the same undersampling percentage using DLMRI, TBMDU, and WTBMDU. Generally, the improvements gained by WTBMDU over other methods are different for four kinds of trajectories although under the same undersampling rate. The largest and smallest improvements were achieved with the 2D random and radial sampling, respectively, where roughly 5 dB and 2 dB were obtained. This indicates that the efficiency of dictionary learning methods may depend on the incoherence of the data acquisition. In the family of WTBMDU algorithms, the optimal *p* value is also different at various trajectories for both WTBMDU-L1 and WTBMDU-L2. To balance the numerical calculation and the selection of trajectories, *p* = 0.5 or *p* = 0.7 is a good option.

**Figure 3:**
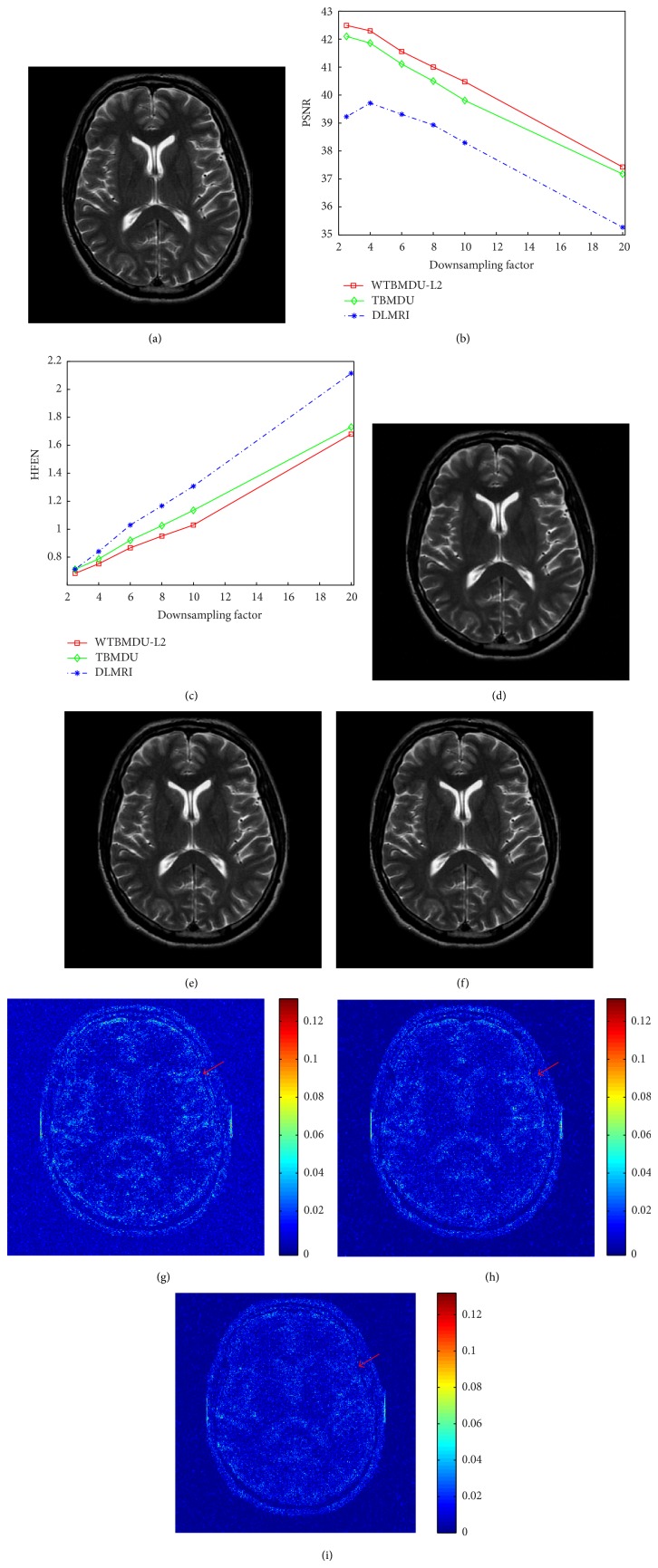
(a) The reference image. ((b) and (c)) The PSNR and HFEN value versus undersampling factor for three methods DLMRI, TBMDU, and WTBMDU at 2.5-, 4-, 6-, 8-, 10-, and 20-fold undersampling. ((d), (e), (f) and (g), (h), (i)) Reconstructions and corresponding error magnitudes for DLMRI, TBMDU, and WTBMDU at 10-fold undersampling, respectively.

Figure 3 illustrates the performance of DLMRI, TBMDU, and WTBMDU at a range of acceleration factors including 2.5, 4, 6, 8, 10, and 20, where zero-mean complex white Gaussian noise with standard deviation *σ* = 10.2 was added to the 2D random sampled* k*-space [29]. Since the stopping rule for the outer loop of both TBMDU and WTBMDU is determined by ||*F*_*p*_ *u* ^*k*^−*f*||_2_ < *σ*, the number of outer iterations *k*_max⁡_ of TBMDU and WTBMDU-L2 with *p* = 0.7 take the values of 3, 3, 6, 7, 7, 11 and 4, 8, 8, 10, 11, 11 for the above six acceleration factors, respectively. For the quantitative comparison, the values of PSNR and HFEN as functions of acceleration factor are shown in Figures 3(b) and 3(c). It can be seen that WTBMDU performs best at all tested acceleration factors. The reconstruction results and the corresponding error images under 10-fold acceleration using three methods are displayed in Figures 3(d), 3(e), and 3(f) and 3(g), 3(h), and 3(i), respectively.

As shown in the error images, the brighter the error image appears, the larger the deviation between the reconstruction and the reference image will be. WTBMDU (Figure 3(i)) presents less pixel errors and structure loss than that of DLMRI in Figure 3(g) and TBMDU in Figure 3(h), especially in regions indicated by red arrows. In general, it can be concluded that both WTBMDU-L2 and TBMDU offer strong preservation of details compared with that of DLMRI, while WTBMDU-L2 provides a crisper result of fine structures than that of TBMDU.

**Figure 4:**
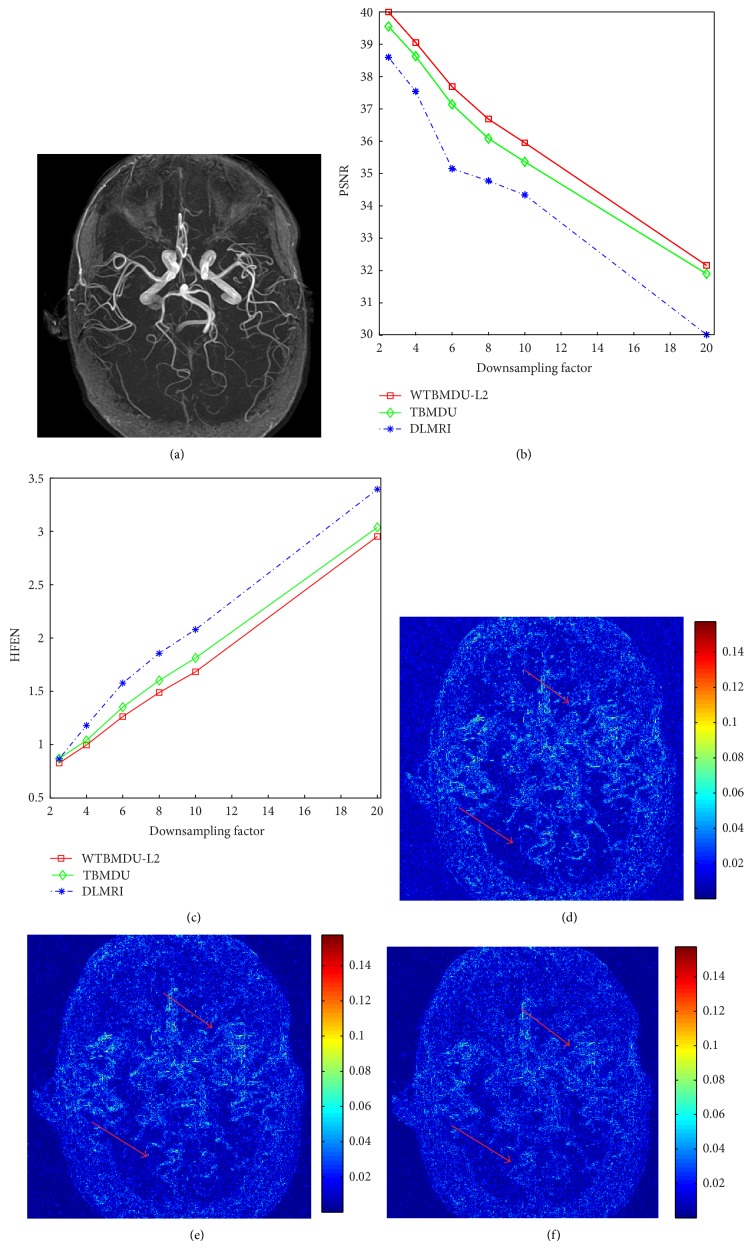
(a) The reference noncontrast MRA of the Circle of Willis. ((b) and (c)) The PSNR and HFEN values versus acceleration factor for the three methods DLMRI, TBMDU, and WTBMDU at 2.5-, 4-, 6-, 8-, 10-, and 20-fold acceleration. ((d), (e), and (f)) Reconstruction error magnitudes for DLMRI, TBMDU, and WTBMDU at 10-fold acceleration, respectively.

Figure 4 depicts the reconstruction results of a transverse slice of a noncontrast MR angiography (MRA) of the circle of Willis (COW) at the same experiment setting as in Figure 3. The MRA of the circle of Willis has much textural information such as the vessels on the middle region and fine-scale details on the bottom region. As can be seen from Figure 4(b), when the acceleration factor increased until 10-fold, the PSNR gap between TBMDU and WTBMDU increases synchronously. The PSNR improvement is also reflected in Figures 4(e) and 4(f) where much less errors appeared in the WTBMDU error image, indicating WTBMDU performs better in maintaining fine details. On the other hand, similar as observed in Figure 3, when the acceleration factor increased to as large as 20-fold, the advantage of the nonconvex optimization degraded and none of these methods can faithfully reconstruct the original image from such fewer* k-*space samples with additional noise.

**Figure 5:**
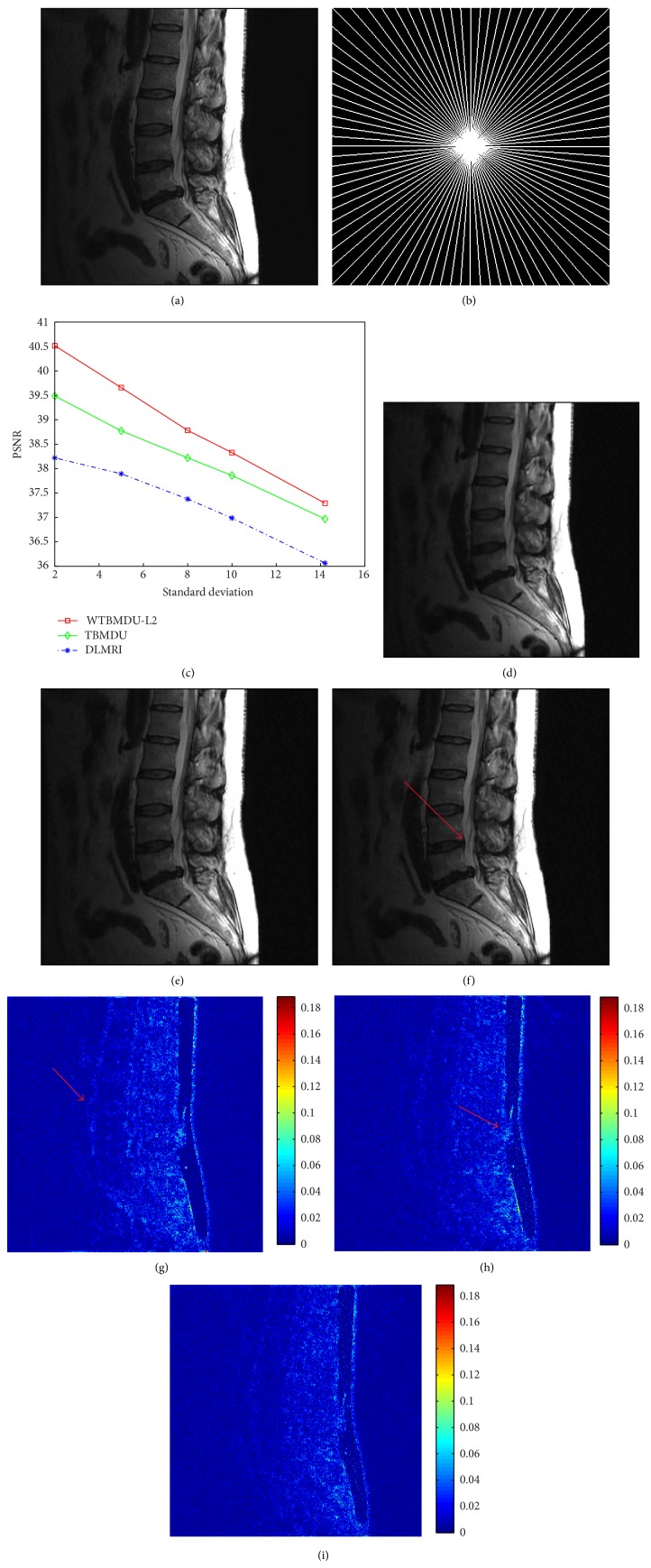
(a) The reference T2-weighted sagittal view of the lumbar spine. (b) Sampling mask in* k*-space with 6.09-fold undersampling. (c) PSNR versus noise level for DLMRI, TBMDU, and WTBMDU. ((d), (e), (f) and (g), (h), (i)) Reconstructions and corresponding error magnitudes for DLMRI, TBMDU, and WTBMDU with noise *σ* = 8.

To investigate the sensitivity of various methods to different levels of complex white Gaussian noise, DLMRI, TBMDU, and WTBMDU were applied to reconstruct a T2-weighted sagittal view of the lumbar spine under pseudo radial sampling at 6.09-fold acceleration.  Figure 5(c) plots the PSNR values of the recovered MR images by DLMRI (blue curves), TBMDU (green curves), and WTBMDU (red curves) at a sequence of different standard deviations (*σ* = 2, 5, 8, 10, 14.2). In the case of *σ* = 2, the PSNR of the image obtained by DLMRI is only 38.22 dB, TBMDU is 39.49 dB, and WTBMDU-L2 with *p* = 0.7 reaches 40.52 dB. Obviously, the difference gap between three methods is significant at low noise levels. The reconstructions and corresponding magnitudes of the error images with *σ* = 8 are shown in Figures 5(d), 5(e), 5(f), 5(g), 5(h), and 5(i). It can be observed that the skeletons in the top half part of the TBMDU reconstruction appear less obscured than those in the DLMRI results. Meanwhile, the reconstruction by WTBMDU is clearer and sharper than that by DLMRI and TBMDU and is relatively devoid of aliasing artifacts. This reveals that our method provides a more accurate reconstruction of image contrast and sharper anatomical depiction in noisy case.

### 4.2. Reconstruction of Experimental MRI Data (Complex-Valued)

**Figure 6:**
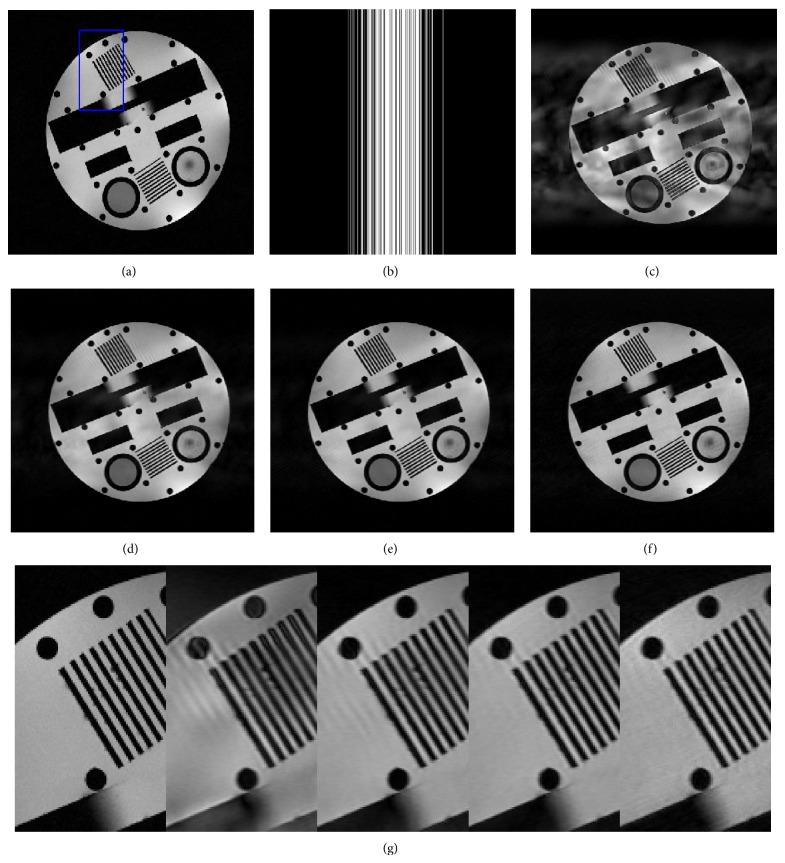
Reconstruction comparison on the real brain data. (a) Reference image corresponding to fully sampled reconstruction. (b) Fourier domain sampling pattern. ((c), (d), (e), and (f)) Reconstruction using DLMRI, TBMDU, WTBMDU-L1, and WTBMDU-L2 with *p* = 0.7 at 80% undersampling, respectively. (g) Enlargements of (a), (c), (d), (e), and (f).

Figure 6 shows the comparison between different methods on a physical phantom, which is often used to assess the resolution of MR reconstruction.  Figure 6(a) displays the fully sampled reconstruction of the physical phantom. Figures 6(c), 6(d), 6(e), and 6(f) exhibit the results of DLMRI, TBMDU, WTBMDU-L1, and WTBMDU-L2 with *p* = 0.7 at 80% undersampling ratio. The corresponding PSNR values are 18.66 dB, 26.82 dB, 28.89 dB, and 29.12 dB, respectively. We can find that the WTBMDU reconstructions exhibit higher resolution than those with DLMRI and TBMDU and are almost devoid of aliasing artifacts especially in zoomed-in regions.

**Figure 7:**
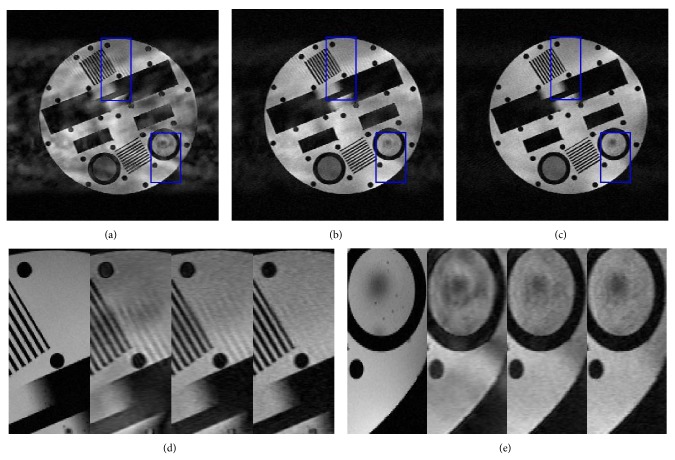
Comparison of reconstructing a physical phantom MR image with noise of *σ* = 30. ((a), (b), and (c)) Reconstructions using DLMRI, TBMDU, and WTBMDU at 5-fold undersampling, respectively. ((d) and (e)) Enlargements of the references (a), (b), and (c).

To investigate the noise sensitivity of proposed method on the complex* k*-space data, complex Gaussian noise of *σ* = 30 was added to the* k*-space with 5-fold acceleration. In this case, the PSNR values of DLMRI, TBMDU, and WTBMDU-L2 with *p* = 0.7 methods are 17.93 dB, 22.94 dB, and 25.42 dB, respectively. The reconstructions of three methods are shown in Figure 7. The enlargements of two region-of-interests (ROIs) are presented in Figures 7(d) and 7(e). As can be observed, the DLMRI reconstruction exhibits more oscillating artifacts than that of the other two methods. Besides, the spot and circle in the bottom right of the WTBMDU reconstruction appear less obscured than those in the DLMRI and TBMDU results.

**Figure 8:**
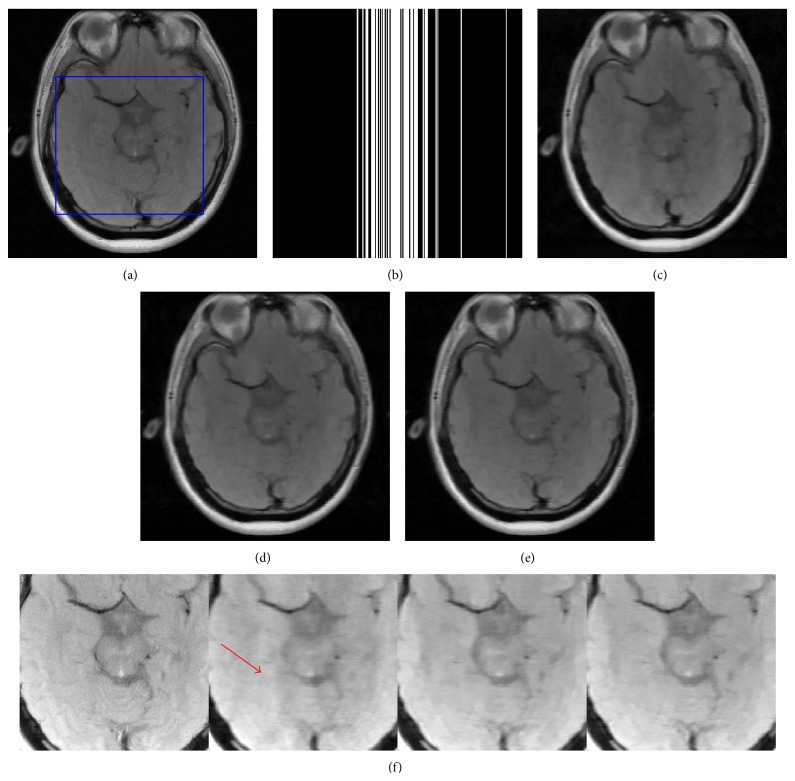
Reconstruction comparison of the real brain data. (a) Reference image corresponding to fully sampled reconstruction. (b) Sampling mask in* k*-space. ((c), (d), and (e)) Reconstruction using DLMRI, TBMDU, and WTBMDU-L2 with *p* = 0.7 at 80% undersampling, respectively. (f) Enlargements of (a), (c), (d), and (e).

**Figure 9:**
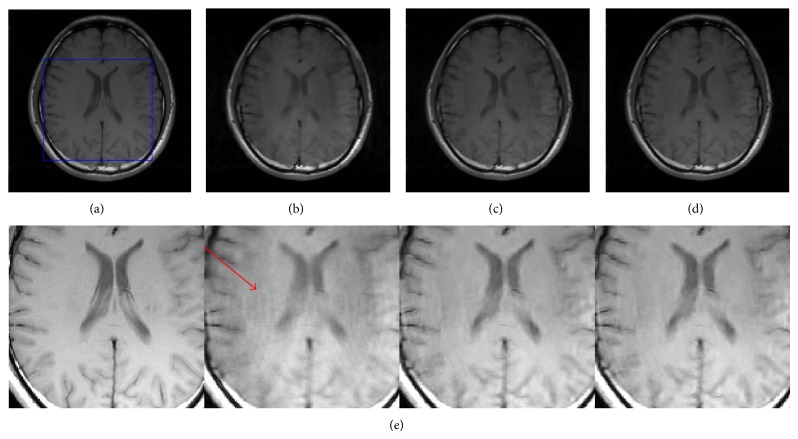
Reconstruction comparison of the real brain data. (a) Reference image corresponding to fully sampled reconstruction. ((b), (c), and (d)) Reconstruction using DLMRI, WTBMDU-L1, and WTBMDU-L2 with *p* = 0.7 at 80% undersampling, respectively. (e) Enlargements of (a), (b), (c), and (d).

In Figures 8 and 9, two real brain data sets containing more fine-detailed structures [44, 46] were used for comparison. The images in Figures 8(a) and 9(a) are both the fully-sampled reconstruction of size 256 × 256 as references. A variable density Cartesian sampling trajectory with 80% undersampling was employed as shown in Figure 8(b). The reconstructions from two data sets using DLMRI, TBMDU and WTBMDU-L2 with *p* = 0.7 are displayed in Figures 8(b), 8(c), and 8(d) and Figures 9(b), 9(c), and 9(d), respectively. The corresponding PSNR values of three methods are 31.35 dB, 32.04 dB, and 32.69 dB for reconstructions in Figure 8 and 30.58 dB, 31.59 dB, and 31.82 dB for those in Figure 9. Some regions of error in these subplots have been illustrated with red arrows, indicating that the reconstruction using WTBMDU shows better fidelity to the reference image than that of DLMRI and TBMDU. The enlargements of the reconstruction results with these methods are shown in Figures 8(f) and 9(e). It can be observed that visible aliasing artifacts along the phase encoding direction (horizontal in the image plane) in the DLMRI reconstruction is more pronounced than those of TBMDU reconstruction, while there are almost no artifacts in that of WTBMDU with *p* = 0.7. Close inspection of the zoomed-in images indicates that the weighted sparse regularization enhances the reconstruction quality.

In summary, the results from our work clearly demonstrate the advantages of introducing iterative reweighting scheme and alternating direction method in nonconvex optimization over conventional methods, for constrained image reconstruction from undersampled* k*-space data. Not only for the brain image containing large piecewise constant regions, as shown in Figures 1, 2, and 3, but also for the circle of Willis and lumbar spine composed of many texture features as shown in Figures 4 and 5, the proposed method visually provides more pleasant results than existing methods. By means of the Bregman iteration/AL methodology, it is notable that the proposed method consistently supports superior results without any parameters tuned manually regardless of different sampling trajectories, varying sampling factors, and the existence of noise or not. We note that, in some circumstances, the PSNR value started to decrease a little bit after achieving the highest value. The reason of this phenomenon is that the solution of problem (1) does not necessarily have higher PSNR than the intermediate iterates. In real-time imaging applications where the acquisition and reconstruction speed is crucial, a numerical algorithm which converges fast at the beginning iterations is highly appreciated and useful.

## 5. Conclusions and Discussions

This work presents a new regularizer combining the nonconvex *l*_*p*_ pseudonorm and adaptive dictionary for MRI reconstruction from undersampled* k*-space data. Based on the potential success of TBMDU in the AL framework for CS-MRI, the proposed WTBMDU method extends the plain TBMDU to handle nonconvex *l*_*p*_ regularization by weighting the sparse coding of the coefficients. The strategy of employing iteratively reweighted *l*_1_ or *l*_2_ technique results in the WTBMDU-L1 and WTBMDU-L2 algorithms, respectively. High-accuracy reconstructions are obtained from significantly undersampled* k*-space data by minimizing the adaptively sparsity-promoting regularization criterion subject to the data-consistency constraint. In particular, compared to the DLMRI method that minimizes the *l*_0_ regularization by the greedy algorithm of matching pursuit, the iteratively reweighted strategy combined with AL framework yields much higher PSNR values. On the other hand, compared to the plain TBMDU for *l*_1_-norm minimization, the adaptively weighted method achieves 0.3–2.5 dB PSNR improvement and visually provides more pleasure results. Various experimental results demonstrate the superior performance of the method under a variety of sampling trajectories and* k*-space acceleration factors.

WTBMDU is very general since it represents an algorithmic framework that can be easily adapted to different reweighting strategies and nonconvex sparsity-inducing functions proposed in the previous literatures. Some extensions will be studied in future as follows: (i) for the reweighted *l*_1_ strategy, using different nonconvex objective functions as conducted in [38, 41]; besides, the shrinkage can be generalized as in [47, 48]; (ii) for the reweighted *l*_2_ strategy, trying different nonconvex objective functions as in [39]. Additionally, the proposed scheme can be easily extended to incorporate other prior (e.g., spike and slab prior used in [49], where the regularization consists of the *l*_2_-norm and *l*_0_-norm terms). It may be better for stabling the numerical process; (iii) the current iteratively reweighted strategies can be combined with the homotopic technique described in [14–16], where the value of *p* slowly decreases from 1 to 0.

Another extension we will consider in future is to extend our proposed model to parallel MRI reconstruction. We note that a very recently published paper [21], under the work of Ramani and Fessler, addressed the regularized SENSE-reconstruction using AL methods. In order to effectively solve the unconstrained SENSE-reconstruction consisting of TV/wavelets regularization term and data-fidelity term using the AL formalism, they split not only the regularization term, but also the Fourier encoding and spatial components in the data-fidelity term, by introducing auxiliary variables to decouple the data-domain components, and the regularization component, respectively. We believe that the regularizer of the sparse representations of overlapping image patches in our work can be naturally incorporated into their work in a unified AL framework.

## Figures and Tables

**Algorithm 1:**
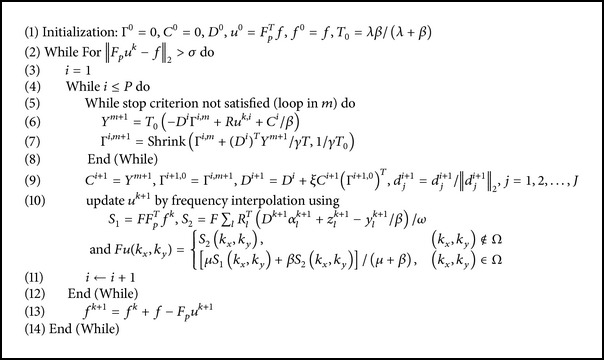
TBMDU.

**Algorithm 2:**
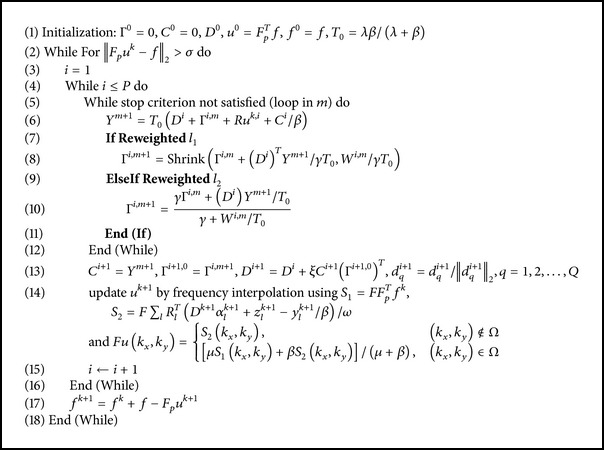
WTBMDU.

**Table 1:** The reconstruction PSNR values of the axial T2-weighted brain image at different sampling trajectories with the same undersampling percentage using different methods.

Sampling mask	2D random sampling	Spiral sampling	Cartesian sampling	Radial sampling
DLMRI	40.54	37.12	35.78	35.32
TBMDU	44.01	38.54	37.41	36.06
WTBMDU-L2, *p* = 0.3	**45.42**	38.31	37.32	36.55
WTBMDU-L2, *p* = 0.5	44.94	38.86	37.61	36.82
WTBMDU-L2, *p* = 0.7	44.78	**39.22**	37.70	36.95
WTBMDU-L1, *p* = 0.3	45.20	38.82	37.62	36.80
WTBMDU-L1, *p* = 0.5	44.88	39.20	37.73	**37.16**
WTBMDU-L1, *p* = 0.7	45.18	39.15	**38.08**	37.06
